# First-Principles Study on the Magnetic Properties of Monolayer MOCl (M = Ti, V, Cr, Mo)

**DOI:** 10.3390/nano16140865

**Published:** 2026-07-14

**Authors:** Yu Pan, Yanjie Wang

**Affiliations:** 1 College of Physics and Electronic Information, Baicheng Normal University, Baicheng 137000, China; peterpancn777@163.com; 2 Key Laboratory for Comprehensive Energy Saving of Cold Regions Architecture of Ministry of Education, School of Electrical and Computer, Jilin Jianzhu University, Changchun 130118, China

**Keywords:** intrinsic ferromagnetic, magnetic anisotropy, first-principles calculations

## Abstract

Two-dimensional (2D) intrinsic ferromagnets with perpendicular magnetic anisotropy (PMA) have been experimentally verified as promising candidates for nanoscale spintronic devices and magnetic random-access memories. In this work, we systematically investigate the stability, electronic structure, and magnetic properties of monolayer MOCl (M = Ti, V, Cr, Mo) via first-principles calculations. The results demonstrate that allshi ciju monolayers MOCl (M = Ti, V, Cr, Mo) are intrinsic ferromagnetic semiconductors, with magnetic moments of 1.0 μ_B_/Ti atom, 2.0 μ_B_/V atom, 2.5 μ_B_/Cr atom and 3.0 μ_B_/Mo atom, respectively. Notably, both monolayers TiOCl and CrOCl exhibit perpendicular magnetic anisotropic energy (MAE), which is mainly contributed by metal atoms Ti and Cr, respectively. Drawing on the second-order perturbation theory, we conduct an analysis of the density of states and the magnetic anisotropy energy (MAE) resolved by d orbitals for Ti and Cr atoms. Our analysis shows that in monolayer TiOCl, the MAE of Ti atoms mainly stems from the disparities in matrix elements between the *d_yz_* and *d*_*x*^2^__−*y*^2^_ (*d_xz_*) orbitals. Conversely, in monolayer CrOCl, the MAE of Cr atoms is largely due to the differences in matrix elements between the *d_xy_* (*d_yz_*) and *d*_*x*^2^__−*y*^2^_ (*d*_*z*^2^_) orbitals. Biaxial strain can efficiently regulate the MAE of monolayer CrOCl. Specifically, when under tensile strain, the MAE of monolayer CrOCl experiences a substantial increase. Our research results indicate that both monolayers TiOCl and CrOCl have significant potential for use in spintronic devices and high-density data storage systems.

## 1. Introduction

Since the successful preparation of graphene and discovery of its extraordinary physical properties by Geim and Novoselov in 2004, two-dimensional (2D) materials have attracted tremendous attention in the academic community [1,2,3,4]. Endowed with numerous novel physical and chemical characteristics, two-dimensional materials have been extensively explored in diverse research fields, including optoelectronic devices, lithium-ion batteries, supercapacitors and spintronics [3,5]. The realization of graphene [1] has confirmed that two-dimensional materials can be exfoliated from van der Waals (vdW) layered crystals [6,7]. This has prompted the investigation of a range of novel two-dimensional (2D) materials, including black phosphorus, hexagonal boron nitride (*h*-BN), and transition metal dichalcogenides (TMDs) [7,8]. As typical two-dimensional materials, these systems exhibit high electrical conductivity, ultrafast charging performance, excellent mechanical strength and rich electronic properties, rendering them crucial for various applications and driving remarkable research progress [2,6]. However, most reported two-dimensional materials are intrinsically nonmagnetic (NM) or weakly magnetic, which largely restricts their further development and application in the field of spintronics [8,9,10]. Ferromagnetic (FM) semiconductor was reported, realizing long-range magnetic order in low-dimensional systems and igniting extensive research interest in novel two-dimensional magnetic materials [1,9,10,11,12,13,14]. The two-dimensional magnets have combined conventional semiconducting and ferromagnetism behavior, and thus offer great potential in the fabrication of high-density information storage and spintronic devices [15,16,17]. Spintronics utilizes both the charge and spin degrees of freedom of electrons, featuring faster processing speed, higher stability and higher storage density [4,8,18,19,20]. Due to these exceptional characteristics, a growing number of scholars have dedicated themselves to the experimental creation and theoretical forecasting of two-dimensional ferromagnetic crystals that possess unique physical attributes, such as magnetic transition metal dichalcogenides, magnetic insulators and magnetic topological insulators [1,18,21]. Nevertheless, the intrinsic non-magnetism of most two-dimensional materials still limits their practical applications in related fields [6]. Magnetism can be introduced into two-dimensional materials through various strategies, including charge doping, adatom adsorption, substitutional doping, defect engineering, surface functionalization and size control [6,8,10,22]. But the realization of the precise control of introducing magnetism using the above strategies is usually difficult in the experiment. On the other hand, the miniaturization demand of high-performance ultrathin spintronic devices is crucial for the development of new intrinsic two-dimensional FM semiconductors [9,10,12,18,23]. According to the Mermin–Wagner theorem, strong thermal fluctuations make long-range magnetic ordering impossible in strictly two-dimensional isotropic Heisenberg systems. Magnetic anisotropy (MA) is therefore essential to suppress thermal fluctuations and stabilize long-range magnetic order in two-dimensional materials [13,22,23]. Unfortunately, the practical application of existing two-dimensional FM materials is greatly limited by their low Curie temperature (T_C_). For instance, the monolayer CrI_3_ with significant magnetic anisotropy energy has a Curie temperature as low as 45 K [24]. Consequently, it is essential to conduct further exploration for novel two-dimensional ferromagnetic (FM) semiconductors that possess a strong magnetic order and a sufficiently high Curie temperature (Tc) [7]. Monolayer chromium oxyhalides CrOX (where X = Cl, Br) have come to the fore as prospective FM semiconductors because of their substantial spin polarization and high Tc [14,23]. In this research paper, we put forward newly emerging two-dimensional intrinsic FM semiconductor materials MOCl (where M = Ti, V, Cr, Mo) through first-principles calculations. The application of mechanical strain has emerged as a powerful tool to tailor the magnetic interactions in two-dimensional materials. For instance, in Fe_3_GeTe_2_-based heterostructures, it was shown that strain, alongside stacking order and electric fields, can significantly modulate both the Dzyaloshinskii–Moriya interaction (DMI) and magnetocrystalline anisotropy energy (MAE), with the tunability arising from a combination of geometric and electronic structure changes [25]. Furthermore, the manipulation of these interactions via strain has direct implications for stabilizing topological spin textures; a notable study demonstrated that compressive strain can lead to the formation of zero-field skyrmions with diameters near 10 nm in a two-dimensional van der Waals heterostructure [26]. These findings provide a strong motivation for our work, where we explore the strain engineering of magnetic interactions in CrOCl.

## 2. Computational Details

All calculations were performed within the framework of the density function theory (DFT) using the projector-augmented plane wave (PAW) potentials, as carried out in the Vienna Ab-initio Simulation Package (VASP) code [27,28]. The exchange-correlation potential was characterized by the generalized gradient approximation (GGA) using the Perdew–Burke–Ernzerhof (PBE) function [29,30]. To accurately account for the strong electron correlation effects of the transition metal M atoms, the GGA + U approach was adopted, with on-site Coulomb interaction parameters U of 7.5 eV assigned to the M-3*d* electrons [31,32]. A cutoff energy of 450 electron volts was utilized for the plane-wave basis. To sample the Brillouin zone, a Gamma-centered Monkhorst-Pack k-point mesh with dimensions of 15 × 17 × 1 was applied. Along the direction perpendicular to the monolayer plane, a vacuum gap of 20 angstroms was established. This was done to avoid any false interactions between the periodic image cells. The lattice constants and all atomic positions were fully optimized using the conjugate gradient method until the residual forces on each atom were less than 0.02 eV Å^−1^ and the energy convergence threshold reached 10^−6^ eV. 

The theoretical foundation of our work rests on the framework of the density function theory. For systems in the presence of external magnetic fields, the standard DFT formalism must be generalized. As established by Grayce and Harris [33], a magnetic field and density function theory (BDFT) can be formulated in which the ground-state energy is a function of both the electron density ρ(r) and the magnetic field B(r). This formalism provides a rigorous theoretical basis for studying the magnetic properties of many electron systems. While our present work employs the more commonly used collinear spin-polarized DFT + U approach without an explicit external magnetic field, the conceptual connection to BDFT is important; both frameworks share the same underlying philosophy of mapping the interacting many electron problem onto an effective single-particle description, and both require careful treatment of exchange and correlation effects in magnetic systems. The BDFT formalism also highlights the fundamental role of the magnetic field as a basic variable alongside the density, which provides valuable context for our strain-engineering approach to tuning magnetic interactions.

The MAE of monolayer MOCl is calculated using the force theorem, with the spin-orbit coupling (SOC) effect treated as a perturbation [34,35,36]. The MAE was defined as the energy difference between the system with magnetization parallel to the monolayer plane and that with magnetization perpendicular to the plane, i.e., $\mathrm{MAE}=E_{X}{-E}_{Z}$. A positive value of the magnetic anisotropy energy (MAE) signifies that the axis of easy magnetization is orthogonal to the plane of the monolayer (perpendicular magnetic anisotropy). Conversely, a negative value implies that the easy axis is parallel to the plane (in-plane magnetic anisotropy). Drawing on the second-order perturbation theory method put forward by Wang et al. [37], the MAE of M atoms can be formulated as follows:

(1) $$\begin{array}{c} MAE=\sum_{\sigma \sigma^{\prime }}E^{\sigma \sigma^{\prime }}\left(x\right)-E^{\sigma \sigma^{\prime }}\left(z\right)\\ =\sum_{\sigma \sigma^{\prime }}{(2\delta }^{\sigma \sigma \prime }-1)\xi^{2}\sum_{O^{\sigma }U^{\sigma^{\prime }}}E^{\sigma \sigma^{\prime }}\frac{{|\left(O^{\sigma }|L_{z}|U^{\sigma^{\prime }}\right)|}^{2}-{|\left(O^{\sigma }|L_{x}|U^{\sigma^{\prime }}\right)|}^{2}}{E_{u}^{\sigma^{\prime }}-E_{o}^{\sigma^{\prime }}} \end{array}$$

where ξ denotes the SOC constant, $E_{u}^{\sigma^{\prime }}$ and $E_{o}^{\sigma^{\prime }}$ are the energy levels of the unoccupied states with spin <$\sigma^{\prime }(|u^{\sigma^{\prime }}\rangle )$> and occupied states with spins <$\sigma (\langle o^{\sigma }|)$>, respectively. The above equation indicates that the mean absolute error (MAE) of titanium (Ti) and chromium (Cr) atoms in monolayer titanium oxychloride (TiOCl) and chromium oxychloride (CrOCl) is influenced by two important factors. First, there is a non-zero difference in the square of the angular momentum matrix elements of the d orbitals. Second, there is an energy gap between the unoccupied and occupied states. This difference refers to the square of the angular momentum matrix element. <${\left|\langle o^{\sigma }|L_{z}|u^{\sigma^{\prime }}\rangle \right|}^{2}-{\left|\langle o^{\sigma }|L_{x}|u^{\sigma^{\prime }}\rangle \right|}^{2}$> is <${\left|\langle o^{\sigma }|u^{\sigma^{\prime }}\rangle \right|}^{2}$> and is simply called the matrix element difference in the following.

Our MAE calculations are based on the magnetic force theorem using the Green’s function formalism. This approach evaluates the energy difference between different magnetization directions using the unperturbed band structure of the magnetic ground state. While we acknowledge that a general caution exists regarding the smoothness of properties at the exact B = 0 point for itinerant electron systems [38], our analysis is focused on the magnetic ground state. The presence of a finite exchange splitting and magnetocrystalline anisotropy ensures a gap in the excitation spectrum, justifying the application of the perturbative Green’s function method for the studied regime.

## 3. Results

**Figure 1:**
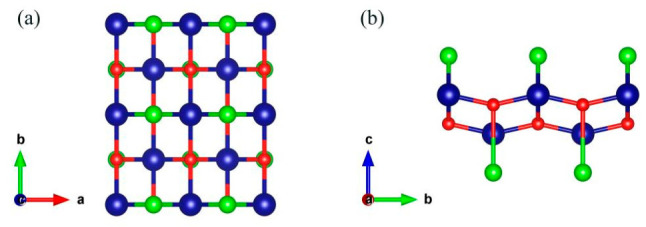
Top (**a**) and side (**b**) views of the crystal structures of monolayer MOCl (M = Ti, V, Cr and Mo).

We first investigated the geometric structure of monolayer MOCl (M = Ti, V, Cr, Mo). As shown in Figure 1, monolayer MOCl adopts an FeOCl-type structure with an orthorhombic space group of *Pmmn*. The crystal structure contains two M atoms, two O atoms and two Cl atoms, which are sandwiched between layers of halogen atoms. The calculated lattice constants of monolayer MOCl are listed in Table 1. It can be observed from Table 1 that the structural constants decrease as the ionic radius of M decreases from Mo to Ti, V, and Cr. Additionally, the calculated lattice constants of monolayer CrOCl are consistent with previous results reported by Qing et al. [31]. To further evaluate the stability and experimental synthesis feasibility of monolayer MOCl, we calculated the binding energies per unit cell using the following equation [39,40]:

$$E_{b}=E_{M}+E_{O}+E_{Cl}-E_{MOCl}$$

where E_MOCl_ is the total energy of a unit cell of monolayer MOCl, E_M_ is the energy of one M atom (Ti V, Cr and Mo), E_o_ is the energy of one oxygen atom and E_Cl_ is the energy of one chlorine atom. The energies of the oxygen and chlorine atoms are taken as the energies of individual atoms in diatomic O_2_ and Cl_2_ molecules, respectively. The calculated binding energies per unit cell are 2.94 eV for monolayer TiOCl, 1.62 eV for VOCl, 1.35 eV for CrOCl and −0.47 eV for MoOCl. The positive binding energies of TiOCl, VOCl and CrOCl indicate their excellent structural stability, and the small negative value of MoOCl also suggests that its synthesis is experimentally feasible.

**Figure 2:**
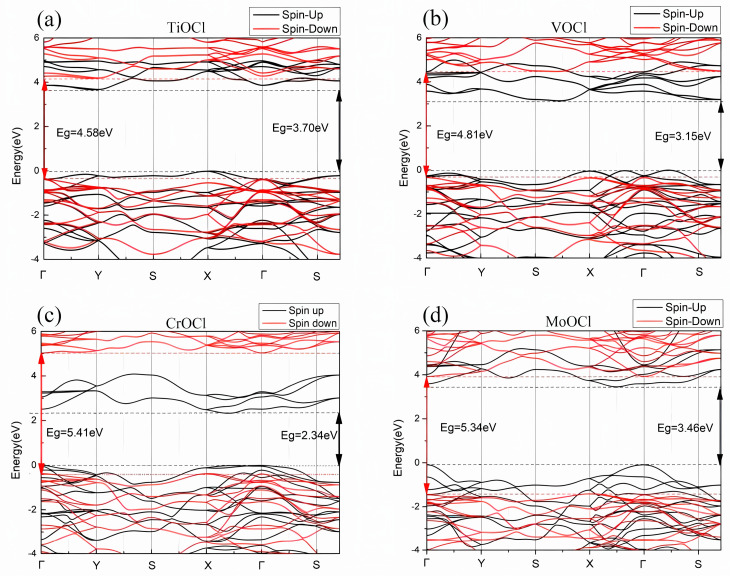
(**a**–**d**) Electronic band structures of monolayer MOCl (M = Ti, V, Cr, Mo). The black and red curves represent the electronic bands spin-up and spin-down, respectively. The Fermi level is set to zero.

As shown in Figure 2, all monolayers MOCl are indirect-band-gap semiconductors with spin-up states dominating the valence band maximum (VMB) and conduction band minimum (CMB) near the Fermi level, indicating significant spin polarization of the electronic structure. The detailed band gap parameters are listed in Table 1. The monolayer CrOCl has an indirect spin-up band gap of 2.34 eV and spin-down band gap of 5.41 eV, where the VBM and CBM occur at the Γ point and along the X-Γ direction, respectively. The monolayer MoOCl has an indirect spin-up band gap of 3.46 eV and spin-down band gap of 5.34 eV, where the VBM and CBM occur at the Γ point and along the X-Γ direction, respectively. The monolayer VOCl has an indirect spin-up band gap of 3.15 eV and spin-down band gap of 4.81 eV, where the VBM and CBM occur along the Γ-S direction and S-X direction, respectively. The monolayer TiOCl has an indirect spin-up band gap of 3.70 eV and spin-down band gap of 4.58 eV, where the VBM and CBM occur at the X point and Γ point, respectively.

To characterize the magnetic ground state of the monolayers MOCl, we calculated the energy difference between the spin-polarized and non-spin-polarized state for monolayer MOCl, defined as ${\Delta E}_{Spin}=E_{sp}-E_{nsp}\;$, where E_sp_ and E_nsp_ are the total energies of the spin-polarized and non-spin-polarized states, respectively. The calculated results are listed in Table 1. The negative ΔE_Spin_ values of monolayers TiOCl, VOCl, CrOCl and MoOCl indicate that their spin-polarized states have lower energy, meaning the magnetic state is the ground state of these monolayers MOCl. The calculated magnetic moments per unit cell of monolayers TiOCl, VOCl, CrOCl and MoOCl are 1.0 μ_B_, 2.0 μ_B_, 2.5 μ_B_ and 3.0 μ_B_, respectively, and the magnetic moments mainly come from metal atoms Ti, V, Cr and Mo atoms.

**Figure 3:**
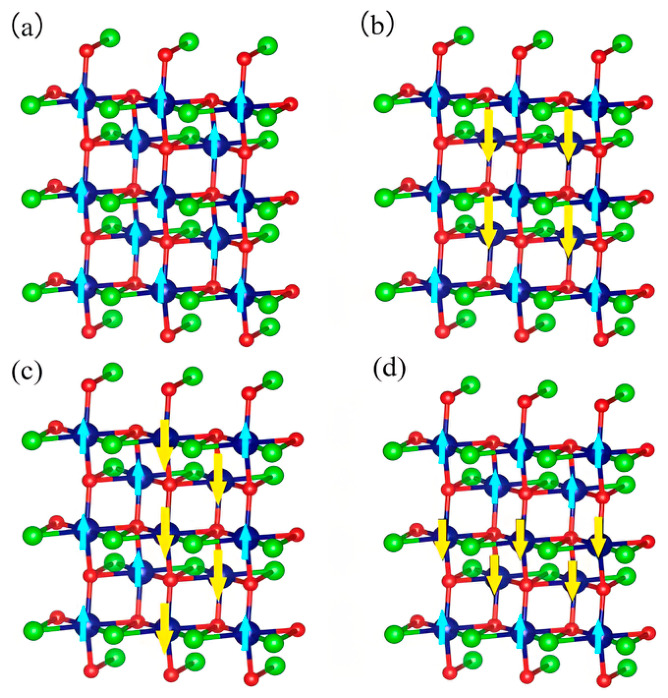
The different magnetic states of monolayer MOCl, where the blue and yellow arrows denote different magnetic moment directions. (**a**) FM, (**b**) AFM-Neel, (**c**) AFM-stripy-1 and (**d**) AFM-stripy-2.

**Figure 4:**
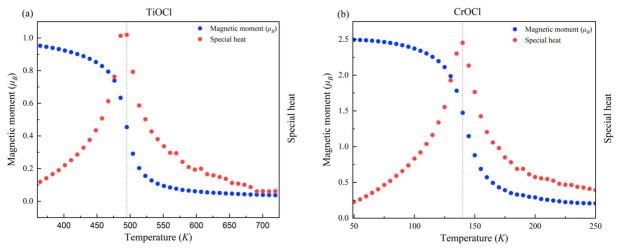
(**a**) The simulated magnetic moment of Ti atom and specific heat in monolayers TiOCl as a function of temperature, (**b**) the simulated magnetic moment of Cr atom and specific heat in monolayers CrOCl as a function of temperature.

To further investigate the magnetic coupling mechanism of monolayer MOCl, we constructed a 2 × 2 × 1 supercell and considered four different magnetic configurations, including the ferromagnetic (FM) state and three anti-ferromagnetic (AFM) states (AFM-Neel, AFM-stripy-1, AFM-stripy-2), as displayed in Figure 3a–d. The energy difference between the AFM and FM states is defined as $\Delta E=-E_{FM}$, and the calculated results are listed in Table 1. A positive ΔE value indicates that the FM state is more thermodynamically stable than the AFM state. The results show that the FM state is more thermodynamically stable than the three AFM states. Accordingly, the Curie temperatures (Tc) of monolayers TiOCl and CrOCl have been calculated as 482 K and 120 K, respectively by using the mean-field theory [39,41,42]. Therefore, both monolayers TiOCl and CrOCl are two-dimensional (2D) intrinsic ferromagnetic (FM) materials. Particularly, the Tc of monolayer CrOCl is much higher than the liquid nitrogen temperature. Additionally, the Curie temperature of TiOCl is greater than the room temperature. To obtain quantitatively reliable estimates of the critical temperatures, we performed classical Monte Carlo simulations using an Ising model with exchange parameters extracted from our DFT + U calculations. For both systems, the magnetic moment decreases gradually as temperature increases, indicative of thermal fluctuation effects. The specific heat, plotted in Figure 4, shows a clear peak at the magnetic ordering temperature. From the position of this peak, the calculated Curie temperature is about 495 K for TiOCl and 139 K for CrOCl [43,44].

**Figure 5:**
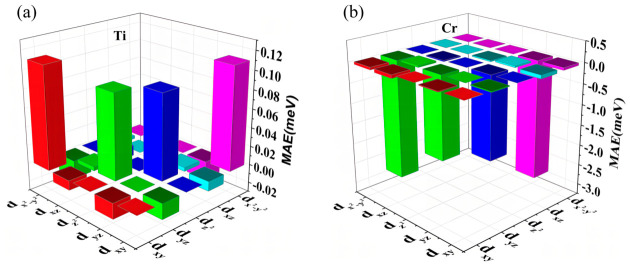
(**a**) The *d*-orbital-resolved MAE of Ti atoms for monolayers TiOCl and (**b**) The *d*-orbital-resolved MAE of Cr atoms for monolayers CrOCl.

Magnetic anisotropy is an important factor to maintain the long-range magnetic order in two-dimensional materials. Therefore, we also studied the magnetic anisotropy energy (MAE) of the MOCl monolayer, and the computed data are shown in Table 1. It should be pointed out that the calculated MAE values of monolayers TiOCl and CrOCl are 0.66 meV per unit cell and 0.4 meV per unit cell, respectively. The positive MAE values indicate their perpendicular magnetic anisotropy (PMA). In order to elucidate the physical basis of the MAE in monolayers TiOCl and CrOCl, we used the second-order perturbation theory to analyze the d-orbital-resolved MAE of Ti and Cr. Figure 5 presents the d-orbital-resolved MAE of Ti and Cr atoms. From Figure 5a, it is clear that the MAE of Ti atoms in monolayer TiOCl is mainly determined by the differences in matrix elements between the *d_yz_* and *d*_*x*^2^__−*y*^2^_ (*d_xz_*) orbitals of Ti, with negative contribution, whereas the contributions from other d orbitals of Ti are much smaller. Figure 5b demonstrates that the MAE of Cr atoms in monolayer CrOCl is mainly due to the differences in matrix elements between the *d_xy_* (*d_yz_*) and *d*_*x*^2^__−*y*^2^_ (*d*_*z*^2^_) orbitals of Cr, with positive contribution. The contributions of MAE from other d orbitals of Cr are obviously less compared with those between the *d_xy_* (*d_yz_*) and *d*_*x*^2^__−*y*^2^_ (*d*_*z*^2^_) orbitals..The disparate orbital contributions to magnetocrystalline anisotropy energy (MAE) from Ti and Cr atoms stem from divergent electron configurations and orbital hybridization behaviors of these two transition-metal ions within the FeOCl-type crystalline framework.

**Figure 6:**
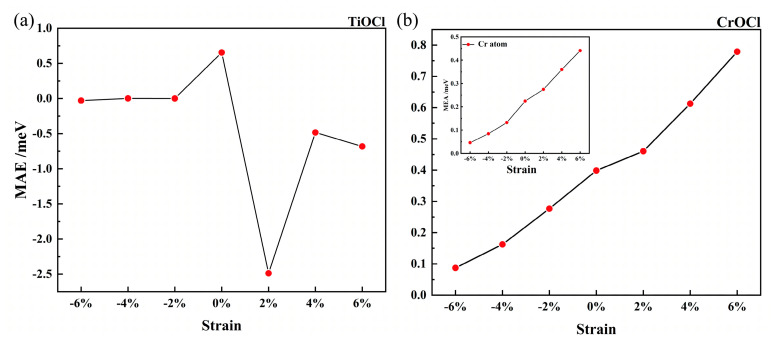
The MAE of monolayers TiOCl (**a**) and CrOCl (**b**). The inset indicates the MAE of the Cr atom.

Next, we investigate the effects of in-plane biaxial strain on the MAE of monolayers TiOCl and CrOCl. The biaxial strain is defined as $\varepsilon =(a-a_{0})/a_{0}$, where a and $a_{0}$ are the strained and unstrained lattice constants of monolayers TiOCl and CrOCl, respectively. Figure 6 gives the MAE per unit cell of monolayers TiOCl and CrOCl under different strains ranging from −6% to 6%. It can be seen from Figure 6a that the MAE of monolayer TiOCl is nearly zero under compressive strain from −2% to −6%, and under tensile strain 2–6%, it changes from PMA to in-plane MAE. This indicates that the biaxial strain cannot control the MAE of monolayer TiOCl because the change in MAE under strain is undesirable. Remarkably, Figure 6b shows that the MAE of monolayer CrOCl changes linearly under biaxial strain. For example, the MAE increases from 0.3 meV per unit cell to 0.8 meV per unit cell under tensile strain from 2% to 6%. While the MAE decreases from 0.3 meV per unit cell to 0.09 meV per unit cell under compressive strain from −2% to −6%. Similar to the unstrained case, the Cr atom contributes the most to the MAE under strain, as shown in the inset in Figure 6b.

**Figure 7:**
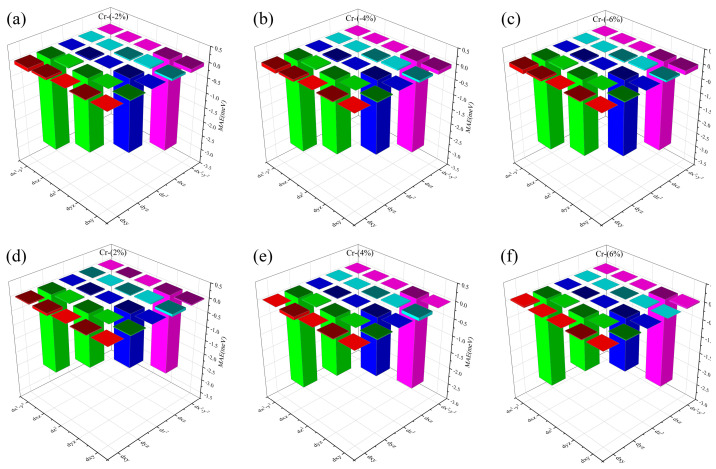
(**a**–**c**) The *d*-orbital-resolved MAE of the Cr atom for monolayer CrOCl under compressive strain from −6% to -2% and (**d**–**f**) the *d*-orbital-resolved MAE of the Cr atom for monolayer CrOCl under tensile strain from 2% to 6%.

To clarify the large MAE of the variation in MAE with strain, we investigate the d-orbital-resolved MAE of Cr atom based on the second-order perturbation theory. Figure 7 presents the d-orbital-resolved MAE of Cr atom under −6%, −4%, −2%, 2%, 4% and 6% strains. As shown in Figure 7a–f, the MAE of Cr atoms in monolayer CrOCl is mainly contributed by the matrix element differences between the *d_xy_* (*d_yz_*) and *d*_*x*^2^__−*y*^2^_ (*d*_*z*^2^_) orbitals of Cr atoms under strain from −6% to 6%. Compared with the unstrained Cr atom of monolayer CrOCl in Figure 5b, the changes in MAE mainly originate from the matrix element differences between the *d*_*x*^2^__−*y*^2^_ (*d*_*z*^2^_) orbitals contribution, as shown Figure 7a–f. And the matrix element differences between the *d*_*x*^2^__−*y*^2^_ (*d*_*z*^2^_) orbitals in Figure 7d–f are obviously smaller than that in Figure 7 a–c. This is the main reason for the increase in MAE of the Cr atom in monolayer CrOCl under tensile strain.

## 4. Conclusions

To summarize, the ground-state configuration of single-layer MOCl is an FeOCl-like structure with the orthorhombic space group Pmmn and the preparation of single-layer MOCl is possible. Additionally, it has been found that both single-layers of TiOCl and CrOCl are two-dimensional (2D) intrinsic ferromagnetic substances with large magnetic anisotropy (MA). Their magnetic moment and MA are mainly attributed to the Ti and Cr atoms. According to the mean-field theory, the Curie temperatures (Tc) of single-layers TiOCl and CrOCl are calculated to be 482 K and 120 K, respectively. Hence, single-layers TiOCl and CrOCl are two-dimensional ferromagnetic materials. The Tc of single-layer CrOCl is much higher than the boiling point of liquid nitrogen and the Tc of TiOCl is above room temperature. By analyzing the d-orbital-resolved magnetic anisotropy energy (MAE), in single-layer TiOCl, the MAE of Ti atoms mainly depends on the differences in matrix elements between the *d_yz_* and *d*_*x*^2^__−*y*^2^_ (*d_xz_*) orbitals of Ti. Similarly, in single-layer CrOCl, the MAE of Cr atoms mainly results from the differences in matrix elements between the *d_xy_* (*d_yz_*) and *d*_*x*^2^__−*y*^2^_ (*d*_*z*^2^_) orbitals of Cr atoms. The biaxial strain can regulate the MAE of monolayer CrOCl, and the MAE of monolayer CrOCl significantly increases under tensile strain. Our results provide a promising route for exploiting two-dimensional intrinsic ferromagnetic materials with large MA and high Curie temperature.

## Figures and Tables

**Table 1:** The optimized lattice constants (a, b), magnetic moments of unit cell, band gap (E_g_), energy difference (ΔE_spin_) between the spin-polarized and non-spin-polarized state, energy difference (ΔE1) between the FM and AFM-Neel state, energy difference (ΔE2) between the FM and AFM-stripy-1 state, energy difference (ΔE3) between the FM and AFM-stripy-2 state of monolayer MOCl (M = Ti, V, Cr, Mo).

MOCl	a (Å)	b (Å)	mag	Band Gap (eV)		ΔE_spin_ (meV)	ΔE1 (meV)	ΔE2 (meV)	ΔE3 (meV)	MAE (meV)
				Spin-Up	Spin-Down					
TiOCl	3.43	4.15	1.0	3.7	4.59	−2.98	30.92	28.02	46.47	0.66
VOCl	3.39	3.96	2.0	3.15	4.82	−7.84	7.92	4.66	6.27	-
CrOCl	3.28	3.98	2.5	2.34	5.41	−7.90	55.0	157.51	54.16	0.40
MoOCl	3.52	4.28	3.0	3.46	5.34	−7.45	31.18	24.83	5.067	-

## Data Availability

The original contributions presented in this study are included in the article. Further inquiries can be directed to the corresponding author.
